# Multiple adaptations to polar and alpine environments within cyanobacteria: a phylogenomic and Bayesian approach

**DOI:** 10.3389/fmicb.2015.01070

**Published:** 2015-10-13

**Authors:** Nathan A. M. Chrismas, Alexandre M. Anesio, Patricia Sánchez-Baracaldo

**Affiliations:** Bristol Glaciology Centre, School of Geographical Sciences, University of BristolBristol, UK

**Keywords:** cyanobacteria, cryosphere, evolution, SSU rRNA gene, phylogenomics, ancestral state reconstruction (ASR)

## Abstract

Cyanobacteria are major primary producers in the polar and alpine regions contributing significantly to nitrogen and carbon cycles in the cryosphere. Recent advancements in environmental sequencing techniques have revealed great molecular diversity of microorganisms in cold environments. However, there are no comprehensive phylogenetic analyses including the entire known diversity of cyanobacteria from these extreme environments. We present here a global phylogenetic analysis of cyanobacteria including an extensive dataset comprised of available small subunit (SSU) rRNA gene sequences of cyanobacteria from polar and high altitude environments. Furthermore, we used a large-scale multi-gene (135 proteins and 2 ribosomal RNAs) genome constraint including 57 cyanobacterial genomes. Our analyses produced the first phylogeny of cold cyanobacteria exhibiting robust deep branching relationships implementing a phylogenomic approach. We recovered several clades common to Arctic, Antarctic and alpine sites suggesting that the traits necessary for survival in the cold have been acquired by a range of different mechanisms in all major cyanobacteria lineages. Bayesian ancestral state reconstruction revealed that 20 clades each have common ancestors with high probabilities of being capable of surviving in cold environments.

## Introduction

Some cyanobacteria can tolerate and even thrive under the extreme conditions found in cold, arid, and UV-exposed environments. They play a key ecological role in many cryo-habitats such as lakes, cryoconites, and lithic substrates (e.g., sandstone and quartz; Quesada and Vincent, 2012), and can be found globally in habitats where temperatures exceed -20 C° (the assumed bottom limit for active metabolism in prokaryotes from natural environments, Clarke et al., 2013) either annually or seasonally. Recent studies that address the evolution of cold cyanobacteria have focused on the mat forming *Microcoleus autumnalis* (= *Phormidium autumnale*; Strunecký et al., 2012a, 2013) and the endolithic *Chroococcidiopsis* sp. (Bahl et al., 2011), but the evolutionary histories of many other lineages remain largely unexplored. Studying the phylogenetic relationships of cyanobacteria from polar and high-altitude environments is essential if we are to understand the mechanisms by which these organisms radiated into such extreme habitats.

Recent advancements in genome sequencing and improved taxon sampling have helped resolve deep branching relationships of the cyanobacteria tree (Sánchez-Baracaldo et al., 2005; Blank and Sánchez-Baracaldo, 2010; Larsson et al., 2011; Shih et al., 2013; Bombar et al., 2014). Large-scale multi-gene phylogenetic analyses have begun converging on similar topologies (Shih et al., 2013; Bombar et al., 2014; Sánchez-Baracaldo et al., 2014) and now provide a robust framework in which to investigate cyanobacterial evolution. Several studies have proposed connections between the diversification of cyanobacteria and major global biogeochemical transitions recorded in the fossil record (Blank and Sánchez-Baracaldo, 2010; Schirrmeister et al., 2013; Sánchez-Baracaldo et al., 2014). While cyanobacterial genomes have helped clarify deep-branching relationships necessary for such analyses, our understanding of the role that cold-tolerant cyanobacteria might have performed in global change has been hindered by an absence of genomes from cold environments. The majority of sequence data available for cyanobacteria from cold habitats are restricted to the small subunit (SSU) rRNA gene (otherwise known as 16S RNA) sequenced direct from environmental samples. While the use of the SSU rRNA gene as a conserved phylogenetic marker has advanced our understanding of microbial communities, its application can be limited when resolving phylogenetic relationships of early divergent microorganisms such as cyanobacteria (Sánchez-Baracaldo et al., 2005) because multiple substitutions at individual loci over long periods of time can lead to false tree topologies when single genes are used, regardless of the number of sequences (Philippe et al., 2011). Furthermore, the ability of short reads to accurately resolve phylogenetic relations has been called into question (Huse et al., 2008; Liu et al., 2009; Youssef et al., 2009) and reliance upon fragmented SSU rRNA gene sequences from environmental samples alone can be problematic. Previous studies of cold filamentous cyanobacteria have treated paraphyletic groups as monophyletic (Casamatta et al., 2005; Strunecký et al., 2010) resulting in misleading interpretations of their evolutionary histories. While Jungblut et al. (2010) and Martineau et al. (2013) employed broader taxon sampling, the extent of the global diversity recovered remains unclear, with poorly resolved relationships between clades.

A key aim of understanding the evolution of cyanobacteria in the cryosphere is to identify cold-specific lineages and ecotypes. One method for predicting cold adaptation involves analyzing the G–C content of rRNA stems, which varies with optimal growth temperature (Galtier et al., 1999). However, since the majority of cyanobacteria from the cryosphere are mesophiles rather than true psychrophiles (Tang et al., 1997), such an approach is unlikely to be informative. Other methods of inferring ancestral characteristics that use genome wide G–C content or amino acid composition (Boussau et al., 2008) require full genomes (or at least multiple proteins) from within a clade of interest. The current lack of complete genomes from cold-tolerant cyanobacteria prohibits such approaches. To compound this, incomplete taxon sampling can introduce biases when interpreting diversity in particular environments. One way to address this is perform analyses independent of these factors. By using ancestral state reconstruction (ASR; Pagel et al., 2004) it is possible to infer the probable habitat preference of the most recent common ancestor (MRCA) of a given clade. This is done by using the length of branches on a phylogenetic tree combined with information about the order in which lineages diverge, and can be used even if few sequences are available.

By using an up-to-date genome constraint (Bombar et al., 2014) deep branching relationships can be enforced (Sánchez-Baracaldo et al., 2005, 2014; Blank and Sánchez-Baracaldo, 2010) on the SSU rRNA gene sequences isolated from the cryosphere. The use of this genome constraint resulted in a robust tree topology, thus presenting a clearer representation of the evolutionary history of cyanobacteria from the cryosphere while confirming that cold-tolerant cyanobacteria are found throughout the tree of life of cyanobacteria. By reconstructing the most probable ancestral habitat of clades containing cold-tolerant cyanobacteria, 20 lineages are shown to have likely adapted to cold environments.

## Materials and Methods

### Construction of SSU rRNA Dataset

**FIGURE 1:**
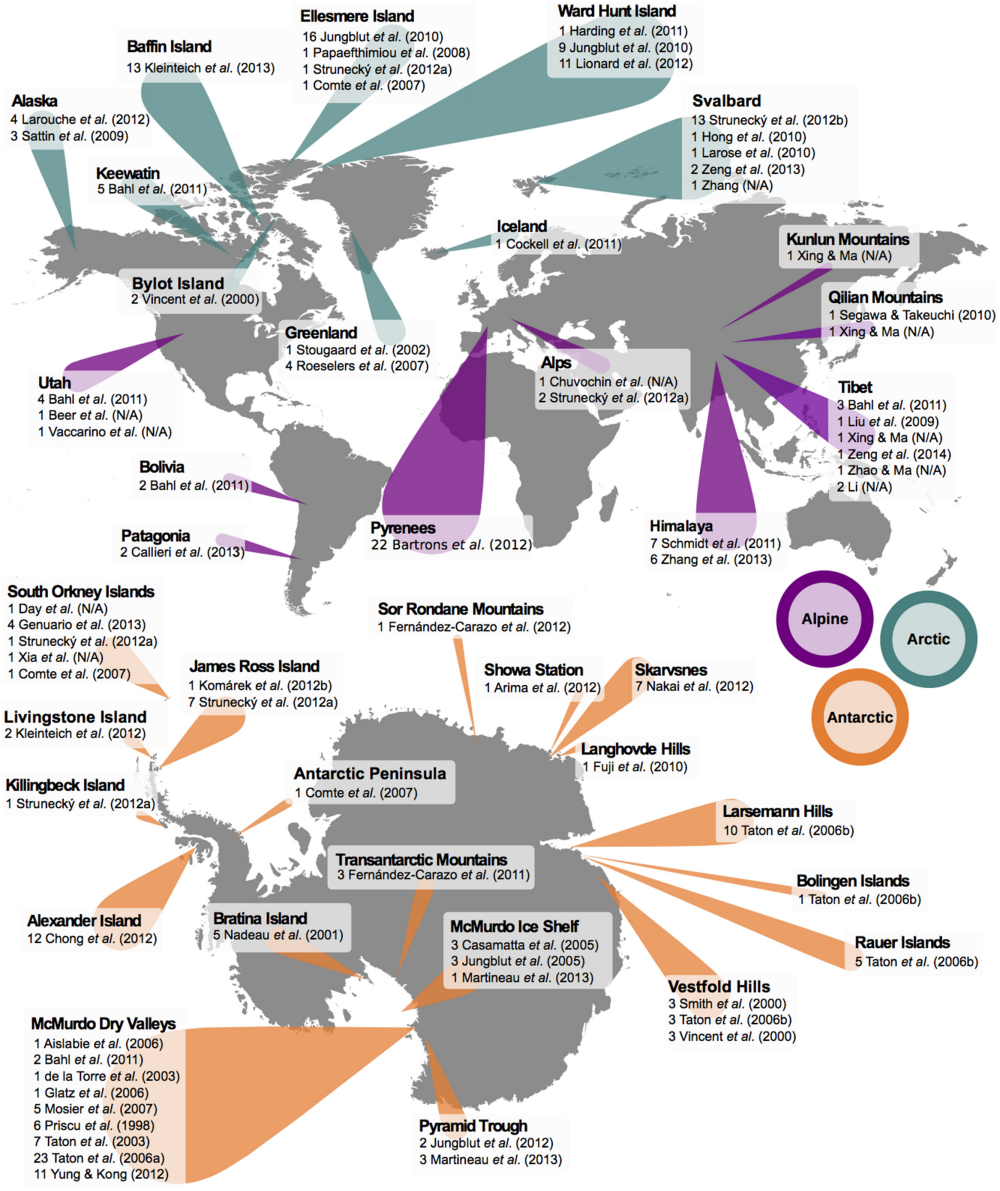
**Geographical locations of cyanobacterial SSU rRNA gene sequences from cold environments used in this study.** Original study and number of sequences from each are shown. Markers indicate either Arctic (blue), Antarctic (orange), or alpine (purple) sampling sites; marker size scales approximately with number of sequences found. Sequences marked N/A are deposited on NCBI GenBank as ’Unpublished’ with no associated journal reference. See Supplementary Tables S1–S3 for further details.

To construct the dataset of cyanobacterial SSU rRNA genes, an extensive search of the literature was carried out to identify previous studies covering cyanobacterial diversity. This provided a basic dataset of well-documented SSU rRNA gene sequences isolated from cold environments (**Figure 1**). In order to expand upon this, naïve BLAST searches (i.e., no sequence information) were then performed with the search terms ‘cyanobacteria’, ‘16S’ ‘SSU’, and combinations of location (e.g., ‘arctic’, ‘antarctic’, and ‘alpine’) and environment (e.g., ‘snow’, ‘ice’, ‘cryoconite’, and ‘cold’). Sequences yielded in this manner were then used as a query for further BLAST searches to uncover more sequences that were less explicitly identified. By doing so, a far larger dataset was built than would have been returned by standard BLAST searches alone. In addition, sequences of temperate and arid origin featuring >92% sequence identity to cryosphere-derived cyanobacteria were also kept. Despite the large range of short reads from environmental studies available, only ‘full length’ SSU rRNA gene sequences were used (*n* = 440, ∼1400 bp) where possible, although some shorter sequences (*n* = 76, ∼700 bp) were allowed due to the relevance of their original publication (such as previous studies into diversity of cyanobacteria in the cryosphere, e.g., Jungblut et al., 2010). Even with these criteria, many sequences had not been assigned species identity and were classified only as ‘uncultured cyanobacteria’. A total of 516 sequences were collated in this manner: 144 from Antarctica, 89 from the Arctic, 60 from alpine environments, and further 223 from other environments. To the above dataset we added the combined SSU rRNA gene sequences used in the phylogenomic studies carried out by Bombar et al. (2014) and Sánchez-Baracaldo et al. (2014) based upon an original dataset compiled in Blank and Sánchez-Baracaldo (2010). Out of a total of 89 sequences, 32 were exclusive to Sánchez-Baracaldo et al. (2014), 22 were exclusive to Bombar et al. (2014), and 35 were shared between datasets. Overall, this generated a final dataset consisting of 605 sequences representing a diverse cross-section of cyanobacterial lineages from both cold and temperate environments. Details of all sequences obtained from cold environments are shown in Supplementary Tables S1–S3. Where no specific journal reference is available, the name associated with the GenBank submission is supplied.

### Sequence Alignment

Alignments were carried out in SATé 2.2.7 (Liu et al., 2009) [using MAFFT (Katoh and Standley, 2013), MUSCLE (Edgar, 2004), and FASTTREE with the CAT approximation (Price et al., 2010)]. Decomposition strategy was set to longest to compensate for long-branch attraction (LBA) and run with a stopping rule of three iterations without score improvement. SATé ran for a total of eight iterations with a final maximum likelihood (ML) score of -110335.388. The alignment was checked in Mesquite 3.01 (Maddison and Maddison, 2015) and trimmed to 1712 characters to remove poorly aligned regions. Trailing gaps were converted to missing data.

### Phylogenetic Analysis

Phylogenies were reconstructed using the CIPRES (Miller et al., 2010) implementation of RAxML-HPC2 on XSEDE 8.1.11 (Stamatakis, 2014). Best model fit as determined by jModelTest 2.1.6 (Darriba et al., 2012) was found to be the general time-reversible model (GTR) with a gamma distribution (G) and a proportion of invariable sites (I) according to the Akaike information criterion (AIC). Despite showing improved model fit, the GTR + G + I model has been demonstrated to be non-identifiable on a tree (Allman et al., 2008); in light of this the GTR + G model was used. A genome constraint was applied in the form of a 57 taxa phylogenomic tree (Bombar et al., 2014) reconstructed using a concatenated alignment of 135 proteins and 2 ribosomal RNAs (see Blank and Sánchez-Baracaldo, 2010 for complete list of sequences used). This tree represents a broad taxonomic sampling across major clades from the cyanobacterial tree. Automatic bootstopping was applied using majority rule criterion and the bootstrap search was automatically halted after 400 replicates. RAxML completed with a final ML optimization likelihood of -66816.485. Trees were checked using FigTree 1.4.0^1^ and annotated using the EvolView web interface^2^ (Zhang et al., 2012). Graphical alterations were done manually using InkScape 0.48^3^.

### Ancestral State Reconstruction

To establish the possibility of past habitat preference for cold environments in specific cyanobacterial lineages, ASR was performed using BayesTraits 2.0 (Pagel et al., 2004)^4^. A truncated version of the initial dataset was created to include closely related sequences from independent habitats while removing near identical sequences from geographically close locations, leaving a total of 270 sequences. Phylogeny was then reconstructed as described previously. The BayesMultiState program (Pagel et al., 2004) of BayesTraits 2.0 was implemented using the single best ML tree with each sequence coded as either cold (C) or other (O). To determine the probabilities that lineages shared a cold-tolerant MRCA, analyses were performed on monophyletic clades containing at least two sequences from cold environments using reversible jump MCMC (RJ-MCMC). To assist in choice of priors, an initial ML analysis was run to determine expected transition rates; an exponential prior drawn from a uniform hyperprior with an interval of 0–1 was chosen as providing the best fit for the data. Rate deviation was set to 10 to maintain acceptance values between 0.2 and 0.4. Analyses were run for a total of 5050000 iterations sampling every 1000 and discarding the first 100000 as burnin. Confidence in each probability was determined by calculating Bayes factors (BFs) from the harmonic means of a pair of analyses in which the node of interest was fixed to either state C or state O (BF < 2 = weak support, BF of 2–5 = positive support, BF of 5–10 = strong support, and BF > 10 = very strong support; see BayesTraits 2.0 documentation). For all analyses, the average was taken from three independent runs to account for variability between runs.

## Results

### Phylogenomic Constraint

**FIGURE 2:**
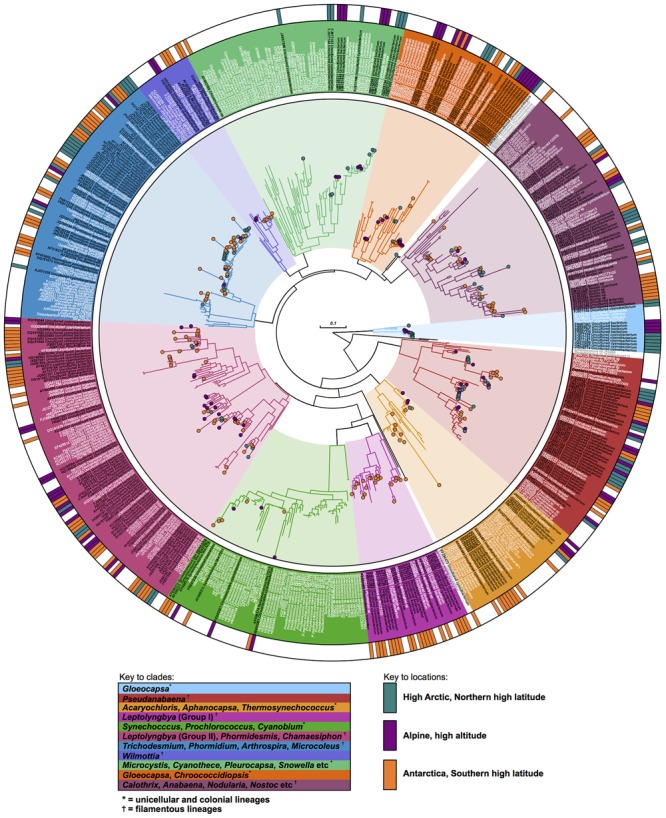
**Phylogeny of cyanobacteria in the cryosphere.** The phylogenetic tree was estimated in a two-step process. First, a genome constraint with 57 taxa was generated using 135 proteins and 2 ribosomal RNAs. Second, a broader taxa sampling including sequences from cold environments was achieved for an additional dataset using SSU rRNA gene sequences and by enforcing the cyanobacteria genome tree in step one (RAxML).

The use of a genome constraint has considerable effect on the overall tree topology. Phylogenetic analyses based on SSU rRNA alone generate misleading phylogenetic relationships for *Pseudanabaena, Aphanocapsa*, and *Chroococcidiopsis* (see Supplementary Figure S1). The genome constraint has helped to resolve the evolutionary histories of these lineages (**Figure 2**).

### Multiple Origins of Cold-Tolerant Clades within Cyanobacteria

**FIGURE 3:**
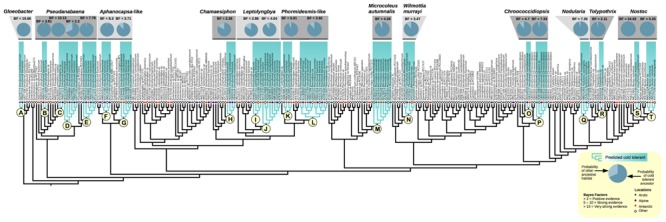
**Ancestral state reconstruction (ASR) of past habitat preference in cyanobacteria found in the cryosphere.** Sequences were assigned a binary character state of cold (Arctic, blue diamonds; alpine, purple diamonds; Antarctic, orange diamonds), or other (white diamonds). Letters A–T denote clades predicted to have a cold-tolerant most recent common ancestor (MRCA) based upon Bayes factor (BF) analysis.

Through out the cyanobacterial tree, BFs support evolutionary shifts enabling some lineages the ability to cope with low temperatures characteristic of polar and alpine regions. ASR revealed 20 lineages with putative cold-tolerant ancestors (**Figure 3**). In the basal group containing the temperate *Gloeobacter violaceus* PCC7421, the majority of sequences from the cryosphere were found in a clade sister to temperate *Gloeobacter* and showed very strong support for a cold-tolerant ancestor (**Figure 3**, letter A: ASR probability = 0.99, BF = 10.66). *Pseudanabaena* appears common in cold environments, with sequence diversity being broadly distributed over several sub-clades. Four sub-clades showed positive support of having a cold-tolerant ancestor (**Figure 3**, letter B: ASR probability = 0.98, BF = 3.91; letter C: ASR probability = 0.99, BF = 10.13; letter D: ASR probability = 0.68, BF = 2.2; and letter E: ASR probability = 0.98, BF = 7.78). Two clades bearing sequence similarity to *Aphanocapsa* and *Thermosynechococcus elongatus* were found to have support for a cold-tolerant ancestor; one containing sequences from Tibet (strong positive support; **Figure 3**, letter (F): ASR probability = 0.98, BF = 8.3) and a second containing sequences from Antarctica (positive support; **Figure 3**, letter G: ASR probability = 0.87, BF = 3.71). A subclade of *Chamaesiphon subglobosus* had positive support for a cold ancestor (**Figure 3**, letter H: ASR probability = 0.83, BF = 2.28). *Leptolyngbya* (Group II) contained two adjacent clades with putative cold-tolerant ancestors. The clade containing *Leptolyngbya antarctica* had positive support for a cold-tolerant MRCA (**Figure 3**, letter I: ASR probability = 0.83, BF = 2.86) whereas its sister had strong positive support (**Figure 3**, letter J: ASR probability = 0.87, BF = 4.037). There was slight indication of increased probability for a cold-tolerant ancestor to both of these clades combined (ASR probability = 0.62) but this value was poorly supported (BF = 1.21). A clade related to *Phormidesmis/Plectolyngbya* had strong support for a cold-tolerant ancestor (**Figure 3**, letter K: ASR probability = 0.94, BF = 6.01). *Phormidesmis priestleyi* (= *Phormidium priestleyi*; Komárek et al., 2009) was found to be present in Antarctic, Arctic, and Alpine environments and had a high probability of a cold MRCA with positive support (**Figure 3**, letter L: ASR probability = 0.88, BF = 3.92). An abundance of sequences for the well-studied *Microcoleus autumnalis* were recovered from the Arctic and Antarctic while samples of this same taxonomic group from outside the cryosphere were relatively uncommon. The clade showed positive support for a cold-tolerant ancestor (**Figure 3**, letter M: ASR probability = 0.93, BF = 4.29). Sequences corresponding to *Wilmottia murrayi* (= *Phormidium murray*; Strunecký et al., 2011) were recovered from both the Antarctic and Alpine environments with high probability and positive support of a cold-tolerant ancestor (**Figure 3**, letter N: ASR probability = 0.88, BF = 3.92). Two clades of *Chroococcidiopsis* had high probabilities of cold ancestors with positive (**Figure 3**, letter O: ASR probability = 0.97, BF = 4.7) and very positive (**Figure 3**, letter P: ASR probability = 0.96, BF = 7.33) support. Within the Nostocales, the entire *Nodularia* clade had strong support for cold-tolerant ancestor (**Figure 3**, letter Q: ASR probability = 0.95, BF = 7.24) and a sub-clade of *Tolypothrix* had positive support (**Figure 3**, letter R: ASR probability = 0.97, BF = 2.11). Two sub-clades of *Nostoc* were found with very strong (**Figure 3**, letter S: ASR probability = 0.99, BF = 24.02) and strong (**Figure 3**, letter T: ASR probability = 0.99, BF = 5.23) support for a cold-tolerant ancestor.

## Discussion

Molecular ecology studies (**Figure 1**) have shown that putative cold adapted cyanobacteria can be found in different biomes and exhibit a wide range of morphological traits (e.g., unicellular, colonial, or filamentous). Although species inference in cyanobacteria is difficult without ecological and physiological data, and many sequences from environmental samples are unclassified, our analyses revealed that several clades contain sequences almost entirely from cold habitats (**Figure 2**). In 20 cases there is strong statistical support based on BF analysis to suggest that lineages can be traced back to an ancestor that was capable of survival in cold extreme environments (**Figure 3**). Jungblut et al. (2010) explored the possibility of biogeographical connections linking cyanobacteria at both poles; here we build upon that understanding based on the results of this study.

### Arctic and Northern Hemisphere Alpine

Strong biogeographical links exist between the Arctic and Northern Hemisphere Alpine regions and contiguous cold habitats may have existed in geologically recent times during Pleistocene glaciations (Ehlers et al., 2011). An expected consequence of this would be that cold-specific lineages from throughout the Northern hemisphere might cluster together. This prediction appears to hold true for certain taxa (e.g., *Gloeobacter*: **Figure 3**, letter A; *Pseudanabaena*: **Figure 3**, letter C) where it is possible to distinguish clades with multiple sequences from the cold biomes of the non-equatorial Northern hemisphere [i.e., within the 10 °C July isotherm (Vincent and Laybourn-Parry, 2008) and/or at altitudes exceeding 2500 m]. Such clusters may represent relict populations of cold resistant strains that were broadly distributed throughout the Northern hemisphere during the last glacial maximum (LGM).

### Antarctica

It has been proposed that Antarctica may host endemic strains of cyanobacteria due to extensive periods of geographic isolation. Previous candidates for Antarctic endemics have included *Wilmottia murrayi* (**Figure 3**, letter N) and *Phormidesmis priestleyi* (**Figure 3**, letter L) (Taton et al., 2006a; Komárek et al., 2009). However, both have been shown to be present in other cold environments (**Figure 2**) corroborating previous findings by Jungblut et al. (2010) in which taxa presumed to be endemic to Antarctica were also found in the high Arctic. Several lineages may still show some potential for Antarctic-specific endemism including the *Aphanocapsa*-like clade (**Figure 3**, letter G), and *Leptolyngbya* (**Figure 3**, letter I).

Despite these observations, our patchy understanding of cyanobacterial diversity in cold extreme environments makes reliable interpretation difficult. For example, given the widespread geographical distribution of *Gloeobacter* (Mareš et al., 2013) its absence from Antarctica is unclear. Where similar phylogenetic patterns are seen in otherwise cosmopolitan lineages, this may indicate a genuine absence of these groups from particular locations due to: (i) barriers to dispersal, (ii) extinction events; or alternatively could be the result of (iii) incomplete taxon sampling. Efforts to tackle the latter will help to shed light on the possibility of the former two.

### Evolution of Cold-Tolerant Cyanobacteria

The mechanisms by which cyanobacteria first radiated into cold environments are uncertain. While more molecular and ecological data are needed to fully characterize groups that have evolved in response to cold extreme habitats, our Bayesian statistical analyses provide strong support for cold-tolerant ancestors of 20 clades (**Figure 3**) that currently thrive in polar and alpine regions. However, it is not yet known whether the mechanisms allowing for survival in the cold were in place before or after the divergence of lineages. Many of the traits needed for survival in cold environments (e.g., drought tolerance, high UV tolerance, and low light conditions for long periods) are also needed in variety of other habitats such as hot deserts (Quesada and Vincent, 2012), and caves; therefore, organisms that evolved under these conditions may already carry the adaptations necessary to exploit cryo-habitats. A similar process has already been proposed for the colonization of the arctic by angiosperms (Zanne et al., 2014) and the relationship of the putative cold-tolerant sub-clade of *Nostoc commune* (**Figure 3**, letter T) to the hot arid *Nostoc indistinguenda* (Řeháková et al., 2007) is suggestive of a drought-tolerant lineage that expanded into cold environments.

Other lineages appear more likely to have a truly cold adapted ancestor. *Microcoleus autumnalis* (**Figure 3**, letter M) is not only dominant in cold environments worldwide but also represents the only lineage for which true psychrophilic strains (optimal growth temperature <15°C) have been identified (Nadeau and Castenholz, 2000). The high specificity of *Wilmottia murrayi* and *Phormidesmis priestleyi* to cold environments suggests that these species are also independent of temperate relatives, at least at a phylogenetic level. Genomic analyses will shed light as to whether this also extends to functional adaptations.

Another possibility not yet considered is that of an ancient cold-tolerant ancestor which later radiated into more diverse environments. An evolutionary progression such as this might leave a clear phylogenetic signal in the form of basal groups from the cryosphere and derived groups from other environments. Interestingly, such a pattern can be observed in the genus *Nodularia* (**Figure 3**, letter Q). *Nodularia* is a cosmopolitan cyanobacterium typified by high morphological and habitat heterogeneity while displaying characteristically low levels of sequence variability. While these factors have compounded attempts at classification (Řeháková et al., 2014), the position of Antarctic *Nodularia* (*Nodularia quadrata*) as basal to *Nodularia spumigena* appears to be maintained across studies (Taton et al., 2006a; Komárek et al., 2015). Although it may be overhasty to assume a true cold ancestor for *Nodularia*, these relationships appear well supported and the reasons for this intriguing phylogenetic position warrant further analysis.

### Cosmopolitan Strains

The currently accepted model of the evolution of cyanobacteria in the cold assumes cosmopolitan species with broad environmental tolerances allowing for the exploitation of marginally habitable environments (Tang et al., 1997). Indeed, in the case of organisms found throughout the cryosphere but exhibiting weak support for a cold-tolerant ancestor (e.g., *Phormidium sensu stricto, Microcoleus vaginatus*) this seems a likely explanation. Many cold-tolerant lineages as described here have emerged from within otherwise cosmopolitan strains and the extent to which niche adaptation has occurred is likely to vary. The putative cold-tolerant sub-clade related to *Phormidesmis*/*Plectolyngbya* (**Figure 3**, letter K) can be extended to include an isolate from a Greenland thermal spring (accession number DQ431004). Evidence for a cold-tolerant ancestor of this extended clade is inconclusive (ASR probability = 0.5, BF = 0.21), implying a link between the poles independent of strict adaptation to the cold.

It is probable that the true nature behind the evolution of cyanobacteria in the cryosphere results from the interplay of each of these processes and more, the details of which will only become clear with more in depth analyses. For example, the deep branching cold lineages in *Chroococcidiopsis* reported by Bahl et al. (2011) were not so clearly resolved in our analyses. This is likely due to absence of the improved phylogenetic resolution afforded by extending the SSU rRNA sequence to include the intergenic spacer (ITS) and large subunit (LSU) (23S) rRNA gene, and applying such techniques to other taxa will no doubt reveal further complexity. Furthermore, more extensive taxon sampling will likely reveal further cold-tolerant lineages where there are only single sequences used in this analysis (e.g., *Calothrix elsteri*; Komárek et al., 2012b, Komárek et al., 2015).

## Conclusions

Although it is clear that the cryosphere hosts a high diversity of cyanobacteria our knowledge is by no means complete. Previous identification by either morphological characteristics or the SSU rRNA gene alone resulted in inconclusive classification of organisms, and while matters have been significantly improved by recent polyphasic studies (Comte et al., 2007; Strunecký et al., 2010; Komárek et al., 2012a,b), further taxonomic revision will likely be required once cyanobacterial genomes from cold habitats are available. Many of the lineages discussed here have had little or no work towards understanding how these organisms are adapted to extreme cold environments or how they may differ from similar organisms from temperate environments. Genomes of cold-tolerant cyanobacteria are urgently needed to further improve our understanding of how these microorganisms have evolved. The reconciliation of these molecular data with morphological characteristics, ecophysiology, and geographical distributions of cold-tolerant cyanobacteria justifies considerable further investigation in both the laboratory and the field. Furthermore, in depth molecular clock studies combined with reconstructions of past climate are required to ascertain the prevailing environmental conditions under which these lineages might first have appeared. A broad study including speciation events of cold, temperate, tropical, and thermophilic lineages may be appropriate to place the appearance of cold-tolerant cyanobacteria within the context of long-term global climate evolution. In the absence of complete genome sequences, the use of a genome constraint has been shown to allow trees constructed using SSU rRNA genes to retain the topology of the new generation of phylogenomic cyanobacterial trees thus opening up the potential for robust evolutionary studies using existing environmental data.

Regardless of current gaps in our knowledge, the presence of numerous cryosphere-specific groups highlights the fascinating potential for cold cyanobacteria as a model system for investigating evolutionary processes. These include global biogeography and distribution mechanisms, adaptation to environmental extremes and biological responses to climatic change, as well as helping to further our overall understanding of cyanobacteria as important nutrient cyclers in cold extreme environments.

## Conflict of Interest Statement

The authors declare that the research was conducted in the absence of any commercial or financial relationships that could be construed as a potential conflict of interest.

## Footnotes

1 http://tree.bio.ed.ac.uk/software/figtree/

2 http://evolgenius.info/evolview

3 http://www.inkscape.org/

4 http://www.evolution.rdg.ac.uk/BayesTraits.html
