# Supplementary material for: Multiple adaptations to polar and alpine environments within cyanobacteria: a phylogenomic and Bayesian approach
Source: Front Microbiol. 2015 Oct 13;6:1070. doi: 10.3389/fmicb.2015.01070 (PMC4602134; doi:10.3389/fmicb.2015.01070)
Supplement: Supplementary file 1 [file Table_1.DOCX]

***Supplementary Material***

**Phylogeny and diversity of cyanobacteria associated with polar and alpine environments**

**Nathan A. M. Chrismas^1^, Alexandre M. Anesio^1^, Patricia Sánchez-Baracaldo^1*^**

^1^Bristol Glaciology Centre, School of Geographical Sciences, University of Bristol, Bristol BS8 1SS, UK

*** Correspondence:** Dr Patricia Sánchez-Baracaldo, School of Geographical Sciences, University of Bristol, Bristol BS8 1SS, UK

p.sanchez-baracaldo@bristol.ac.uk

**1. Supplementary Tables**

Supplementary Table 1: High altitude and alpine sequences

| Accession | Description | Location | Reference |
| --- | --- | --- | --- |
| FJ546713 | Nodularia spumigena GSL023 16S ribosomal RNA gene, partial sequence | USA Utah | Beer *et al*., N/A |
| FJ805888 | Uncultured Chroococcidiopsis sp. clone NA2_2 16S ribosomal RNA gene, partial sequence; 16S-23S ribosomal RNA intergenic spacer, complete sequence; and 23S ribosomal RNA gene, partial sequence | USA Utah | Bahl *et al*., 2011 |
| FJ805894 | Uncultured Chroococcidiopsis sp. clone NA2_8 16S ribosomal RNA gene, partial sequence; 16S-23S ribosomal RNA intergenic spacer, complete sequence; and 23S ribosomal RNA gene, partial sequence | USA Utah | Bahl *et al*., 2011 |
| FJ805895 | Uncultured Chroococcidiopsis sp. clone NA2_9 16S ribosomal RNA gene, partial sequence; 16S-23S ribosomal RNA intergenic spacer, complete sequence; and 23S ribosomal RNA gene, partial sequence | USA Utah | Bahl *et al*., 2011 |
| FJ805906 | Uncultured Chroococcidiopsis sp. clone NA3_9 16S ribosomal RNA gene, partial sequence; 16S-23S ribosomal RNA intergenic spacer, complete sequence; and 23S ribosomal RNA gene, partial sequence | USA Utah | Bahl *et al*., 2011 |
| JQ083658 | Tolypothrix sp. CNP3-B1-C1 clone p12C 16S ribosomal RNA gene, partial sequence; 16S-23S ribosomal RNA intergenic spacer, tRNA-Ile and tRNA-Ala genes, complete sequence; and 23S ribosomal RNA gene, partial sequence | USA Utah | Vaccarino *et al*., N/A |
| AB569623 | Uncultured bacterium genes for 16S rRNA, ITS, clone: Qiyi-cya-OTU 0 | China Xiongguan | Segawa & Takeuchi, 2010 |
| EU527180 | Uncultured bacterium clone zd1-72 16S ribosomal RNA gene, partial sequence | Tibetan Plateau | Liu *et al*., 2009 |
| FJ805924 | Uncultured Chroococcidiopsis sp. clone AS2_1 16S ribosomal RNA gene, partial sequence; 16S-23S ribosomal RNA intergenic spacer, complete sequence; and 23S ribosomal RNA gene, partial sequence | Tibetan Plateau | Bahl *et al*., 2011 |
| FJ805925 | Uncultured Chroococcidiopsis sp. clone AS3_1 16S ribosomal RNA gene, partial sequence; 16S-23S ribosomal RNA intergenic spacer, complete sequence; and 23S ribosomal RNA gene, partial sequence | Tibetan Plateau | Bahl *et al*., 2011 |
| FJ805927 | Uncultured Chroococcidiopsis sp. clone AS3_3 16S ribosomal RNA gene, partial sequence; 16S-23S ribosomal RNA intergenic spacer, complete sequence; and 23S ribosomal RNA gene, partial sequence | Tibetan Plateau | Bahl *et al*., 2011 |
| HM127448 | Uncultured bacterium clone SINP426 16S ribosomal RNA gene, partial sequence | Himalaya | Zhang *et al*., 2013 |
| HM127934 | Uncultured bacterium clone SINH1111 16S ribosomal RNA gene, partial sequence | Himalaya | Zhang *et al*., 2013 |
| HM127934 | Uncultured bacterium clone SINH747 16S ribosomal RNA gene, partial sequence | Himalaya | Zhang *et al*., 2013 |
| HM129015 | Uncultured bacterium clone SING1109 16S ribosomal RNA gene, partial sequence | Himalaya | Zhang *et al*., 2013 |
| HM129043 | Uncultured bacterium clone SING1141 16S ribosomal RNA gene, partial sequence | Himalaya | Zhang *et al*., 2013 |
| HM129552 | Uncultured bacterium clone SING991 16S ribosomal RNA gene, partial sequence | Himalaya | Zhang *et al*., 2013 |
| HQ188993 | Uncultured cyanobacterium clone B10912H 16S ribosomal RNA gene, partial sequence | Himalaya | Schmidt *et al*., 2011 |
| HQ189004 | Uncultured cyanobacterium clone B109212A 16S ribosomal RNA gene, partial sequence | Himalaya | Schmidt *et al*., 2011 |
| HQ189026 | Uncultured cyanobacterium clone B10811G 16S ribosomal RNA gene, partial sequence | Himalaya | Schmidt *et al*., 2011 |
| HQ189027 | Uncultured cyanobacterium clone B10812B 16S ribosomal RNA gene, partial sequence | Himalaya | Schmidt *et al*., 2011 |
| HQ189039 | Uncultured cyanobacterium clone B108207C 16S ribosomal RNA gene, partial sequence | Himalaya | Schmidt *et al*., 2011 |
| HQ189044 | Uncultured cyanobacterium clone B108208E 16S ribosomal RNA gene, partial sequence | Himalaya | Schmidt *et al*., 2011 |
| HQ189080 | Uncultured cyanobacterium clone B107204A 16S ribosomal RNA gene, partial sequence | Himalaya | Schmidt *et al*., 2011 |
| HQ327233 | Uncultured bacterium clone TP-Snow-96 16S ribosomal RNA gene, partial sequence | Tibetan Plateau | Zhao & MA, N/A |
| JN225548 | Uncultured cyanobacterium clone GSY-XJ22 16S ribosomal RNA gene, partial sequence | Tibetan Plateau | Li, N/A |
| JN225549 | Uncultured cyanobacterium clone GSY-XJ26 16S ribosomal RNA gene, partial sequence | Tibetan Plateau | Li, N/A |

Supplementary Table 1 (continued): High altitude and alpine sequences

| Accession | Description | Location | Reference |
| --- | --- | --- | --- |
| JQ327950 | Uncultured bacterium clone ch-xj85 16S ribosomal RNA gene, partial sequence | Tibetan Plateau | Zeng *et al*., 2014 |
| KC286729 | Uncultured bacterium clone gls72 16S ribosomal RNA gene, partial sequence | Xinyang | Xing & Ma, N/A |
| KC286840 | Uncultured bacterium clone gls96 16S ribosomal RNA gene, partial sequence | Tibetan Plateau | Xing & Ma, N/A |
| KC286890 | Uncultured bacterium clone gls238 16S ribosomal RNA gene, partial sequence | China Xiongguan | Xing & Ma, N/A |
| FN984813 | Uncultured bacterium partial 16S ribosomal RNA gene, clone RA_019 | Pyrenees | Bartrons *et al*., 2012 |
| FN984870 | Uncultured bacterium partial 16S ribosomal RNA gene, clone RA_076 | Pyrenees | Bartrons *et al*., 2012 |
| FN984880 | Uncultured bacterium partial 16S ribosomal RNA gene, clone RA_086 | Pyrenees | Bartrons *et al*., 2012 |
| FR667236 | Uncultured bacterium partial 16S rRNA gene, clone Lle_004 | Pyrenees | Bartrons *et al*., 2012 |
| FR667281 | Uncultured bacterium partial 16S rRNA gene, clone Lle_049 | Pyrenees | Bartrons *et al*., 2012 |
| FR667316 | Uncultured bacterium partial 16S rRNA gene, clone Llo_018 | Pyrenees | Bartrons *et al*., 2012 |
| FR667369 | Uncultured bacterium partial 16S rRNA gene, clone Llo_071 | Pyrenees | Bartrons *et al*., 2012 |
| FR667373 | Uncultured bacterium partial 16S rRNA gene, clone Llo_075 | Pyrenees | Bartrons *et al*., 2012 |
| FR667376 | Uncultured bacterium partial 16S rRNA gene, clone Llo_078 | Pyrenees | Bartrons *et al*., 2012 |
| FR667396 | Uncultured bacterium partial 16S rRNA gene, clone Be_014 | Pyrenees | Bartrons *et al*., 2012 |
| FR667399 | Uncultured bacterium partial 16S rRNA gene, clone Be_017 | Pyrenees | Bartrons *et al*., 2012 |
| FR667418 | Uncultured bacterium partial 16S rRNA gene, clone Be_036 | Pyrenees | Bartrons *et al*., 2012 |
| FR667439 | Uncultured bacterium partial 16S rRNA gene, clone Be_057 | Pyrenees | Bartrons *et al*., 2012 |
| FR667441 | Uncultured bacterium partial 16S rRNA gene, clone Be_059 | Pyrenees | Bartrons *et al*., 2012 |
| FR667443 | Uncultured bacterium partial 16S rRNA gene, clone Be_061 | Pyrenees | Bartrons *et al*., 2012 |
| FR667456 | Uncultured bacterium partial 16S rRNA gene, clone Be_074 | Pyrenees | Bartrons *et al*., 2012 |
| FR667486 | Uncultured bacterium partial 16S rRNA gene, clone GBe_013 | Pyrenees | Bartrons *et al*., 2012 |
| FR667505 | Uncultured bacterium partial 16S rRNA gene, clone GBe_032 | Pyrenees | Bartrons *et al*., 2012 |
| FR667531 | Uncultured bacterium partial 16S rRNA gene, clone GBe_058 | Pyrenees | Bartrons *et al*., 2012 |
| FR667540 | Uncultured bacterium partial 16S rRNA gene, clone GBe_067 | Pyrenees | Bartrons *et al*., 2012 |
| FR667547 | Uncultured bacterium partial 16S rRNA gene, clone GBe_074 | Pyrenees | Bartrons *et al*., 2012 |
| FR667551 | Uncultured bacterium partial 16S rRNA gene, clone GBe_078 | Pyrenees | Bartrons *et al*., 20122 |
| JF832304 | Uncultured Chamaesiphon sp. clone 5f-17 16S ribosomal RNA gene, partial sequence | Alps | Chuvochin *et al*., N/A |
| JN230340 | Phormidium cf. autumnale CCALA 145 16S ribosomal RNA gene and 16S-23S ribosomal RNA intergenic spacer, partial sequence | Alps | Strunecký *et al*., 2012a |
| JN230343 | Phormidium setchellianum CCALA 144 16S ribosomal RNA gene and 16S-23S ribosomal RNA intergenic spacer, partial sequence | Alps | Strunecký *et al*., 2012a |
| FJ805922 | Uncultured Chroococcidiopsis sp. clone SA2_1 16S ribosomal RNA gene, partial sequence; 16S-23S ribosomal RNA intergenic spacer, complete sequence; and 23S ribosomal RNA gene, partial sequence | Bolivia | Bahl *et al*., 2011 |
| FJ805923 | Uncultured Chroococcidiopsis sp. clone SA2_2 16S ribosomal RNA gene, partial sequence; 16S-23S ribosomal RNA intergenic spacer, complete sequence; and 23S ribosomal RNA gene, partial sequence | Bolivia | Bahl *et al*., 2011 |

Supplementary Table 1 (continued): High altitude and alpine sequences

| Accession | Description | Location | Reference |
| --- | --- | --- | --- |
| HE805921 | Chroococcales cyanobacterium PE3C6 partial 16S rRNA gene, isolate PE3C6 | Patagonia | Callieri *et al*., 2012 |
| HE805927 | Chroococcales cyanobacterium PE1E11 partial 16S rRNA gene, isolate PE1E11 | Patagonia | Callieri *et al*., 2012 |

Supplementary Table 2: Northern high latitude and Arctic sequences

| Accession | Description | Location | Reference |
| --- | --- | --- | --- |
| AF098373 | Synechococcus-like str. P211 16S ribosomal RNA gene, partial sequence | Bylot Island | Vincent *et al*., 2000 |
| AF098374 | Synechococcus-like str. P212 16S ribosomal RNA gene, partial sequence | Bylot Island | Vincent *et al*., 2000 |
| AJ431339 | Uncultured cyanobacterium partial 16S rRNA gene, isolate ikaite un-c15 | Greenland | Stougaard *et al*., 2002 |
| AM711528 | Nostoc sp. Lukesova 40/93 partial 16S rRNA gene, strain Lukesova 40/93 | Ellesmere Island | Papaefthimiou *et al*., 2008 |
| DQ431001 | Leptolyngbya sp. Greenland_6 16S ribosomal RNA gene, partial sequence | Greenland | Roeselers *et al*., 2007 |
| DQ431002 | Leptolyngbya sp. Greenland_7 16S ribosomal RNA gene, partial sequence | Greenland | Roeselers *et al*., 2007 |
| DQ431004 | Cf. Leptolyngbya sp. Greenland_9 16S ribosomal RNA gene, partial sequence | Greenland | Roeselers *et al*., 2007 |
| DQ431005 | Cf. Leptolyngbya sp. Greenland_10 16S ribosomal RNA gene, partial sequence | Greenland | Roeselers *et al*., 2007 |
| DQ493873 | Phormidium autumnale Arct-Ph5 16S ribosomal RNA gene, partial sequence | Ellesmere Island | Comte *et al*., 2007 |
| FJ805852 | Uncultured Chroococcidiopsis sp. clone AR1_3 16S ribosomal RNA gene, partial sequence; 16S-23S ribosomal RNA intergenic spacer, complete sequence; and 23S ribosomal RNA gene, partial sequence | Arctic Keewatin | Bahl *et al*., 2011 |
| FJ805853 | Uncultured Chroococcidiopsis sp. clone AR1_4 16S ribosomal RNA gene, partial sequence; 16S-23S ribosomal RNA intergenic spacer, complete sequence; and 23S ribosomal RNA gene, partial sequence | Arctic Keewatin | Bahl *et al*., 2011 |
| FJ805854 | Uncultured Chroococcidiopsis sp. clone AR2_1 16S ribosomal RNA gene, partial sequence; 16S-23S ribosomal RNA intergenic spacer, complete sequence; and 23S ribosomal RNA gene, partial sequence | Arctic Keewatin | Bahl *et al*., 2011 |
| FJ805855 | Uncultured Chroococcidiopsis sp. clone AR2_2 16S ribosomal RNA gene, partial sequence; 16S-23S ribosomal RNA intergenic spacer, complete sequence; and 23S ribosomal RNA gene, partial sequence | Arctic Keewatin | Bahl *et al*., 2011 |
| FJ805856 | Uncultured Chroococcidiopsis sp. clone AR2_3 16S ribosomal RNA gene, partial sequence; 16S-23S ribosomal RNA intergenic spacer, complete sequence; and 23S ribosomal RNA gene, partial sequence | Arctic Keewatin | Bahl *et al*., 2011 |
| FJ849078 | Uncultured bacterium clone EpiNCA2 16S ribosomal RNA gene, partial sequence | Alaska | Larouche *et al*., 2012 |
| FJ849139 | Uncultured bacterium clone EpiNCB16 16S ribosomal RNA gene, partial sequence | Alaska | Larouche *et al*., 2012 |
| FJ849213 | Uncultured bacterium clone EpiUMA31 16S ribosomal RNA gene, partial sequence | Alaska | Larouche *et al*., 2012 |
| FJ849316 | Uncultured bacterium clone EpiUMB61 16S ribosomal RNA gene, partial sequence | Alaska | Larouche *et al*., 2012 |
| FJ946524 | Uncultured cyanobacterium clone LSS-F5 16S ribosomal RNA gene, partial sequence | Svalbard | Larose *et al*., 2010 |
| FJ977103 | Uncultured cyanobacterium clone PA127 16S ribosomal RNA gene, partial sequence | Ellesmere Island | Jungblut *et al*., 2010 |
| FJ977106 | Uncultured cyanobacterium clone PA131 16S ribosomal RNA gene, partial sequence | Ellesmere Island | Jungblut *et al*., 2010 |
| FJ977108 | Uncultured cyanobacterium clone PA150 16S ribosomal RNA gene, partial sequence | Ellesmere Island | Jungblut *et al*., 2010 |
| FJ977109 | Uncultured cyanobacterium clone PA153 16S ribosomal RNA gene, partial sequence | Ellesmere Island | Jungblut *et al*., 2010 |
| FJ977111 | Uncultured cyanobacterium clone PA159 16S ribosomal RNA gene, partial sequence | Ellesmere Island | Jungblut *et al*., 2010 |
| FJ977115 | Uncultured cyanobacterium clone PA182 16S ribosomal RNA gene, partial sequence | Ellesmere Island | Jungblut *et al*., 2010 |

Supplementary Table 2 (continued): Northern high latitude and Arctic sequences

| Accession | Description | Location | Reference |  |
| --- | --- | --- | --- | --- |
| FJ977116 | Uncultured cyanobacterium clone PA183 16S ribosomal RNA gene, partial sequence | Ellesmere Island | Jungblut *et al*., 2010 |  |
| FJ977138 | Uncultured cyanobacterium clone QL190 16S ribosomal RNA gene, partial sequence | Ward Hunt Island | Jungblut *et al*., 2010 |  |
| FJ977139 | Uncultured cyanobacterium clone QL193 16S ribosomal RNA gene, partial sequence | Ward Hunt Island | Jungblut *et al*., 2010 |  |
| FJ977144 | Uncultured cyanobacterium clone WHL47 16S ribosomal RNA gene, partial sequence | Ward Hunt Island | Jungblut *et al*., 2010 |  |
| FJ977147 | Uncultured cyanobacterium clone WHL69 16S ribosomal RNA gene, partial sequence | Ward Hunt Island | Jungblut *et al*., 2010 |  |
| FJ977153 | Uncultured cyanobacterium clone WHL77 16S ribosomal RNA gene, partial sequence | Ward Hunt Island | Jungblut *et al*., 2010 |  |
| FJ977155 | Uncultured cyanobacterium clone WHL82 16S ribosomal RNA gene, partial sequence | Ward Hunt Island | Jungblut *et al*., 2010 |  |
| FJ977156 | Uncultured cyanobacterium clone WHL85 16S ribosomal RNA gene, partial sequence | Ward Hunt Island | Jungblut *et al*., 2010 |  |
| FJ977157 | Uncultured cyanobacterium clone WHL86 16S ribosomal RNA gene, partial sequence | Ward Hunt Island | Jungblut *et al*., 2010 |  |
| FJ977158 | Uncultured cyanobacterium clone WHL87 16S ribosomal RNA gene, partial sequence | Ward Hunt Island | Jungblut *et al*., 2010 |  |
| GQ396895 | Uncultured bacterium clone AK4AB1_05A 16S ribosomal RNA gene, partial sequence | Alaska | Sattin *et al*., 2009 |  |
| GQ397056 | Uncultured bacterium clone AK4DE1_09H 16S ribosomal RNA gene, partial sequence | Alaska | Sattin *et al*., 2009 |  |
| GQ397073 | Uncultured bacterium clone AK4DE2_01G 16S ribosomal RNA gene, partial sequence | Alaska | Sattin *et al*., 2009 |  |
| HM241943 | Nodularia spumigena KNUA005 16S ribosomal RNA gene, partial sequence | Svalbard | Hong *et al*., 2010 |  |
| HQ230237 | Cyanobacterium cWHL-10 16S ribosomal RNA gene, partial sequence | Ward Hunt Island | Harding *et al*., 2011 |  |
| HQ595191 | Uncultured cyanobacterium clone IC3007 16S ribosomal RNA gene, partial sequence | Svalbard | Zeng *et al*., 2013 |  |
| HQ622720 | Uncultured bacterium clone IC4002 16S ribosomal RNA gene, partial sequence | Svalbard | Zeng *et al*., 2013 |  |
| JN230345 | Phormidium autumnale CCALA 697 16S ribosomal RNA gene and 16S-23S ribosomal RNA intergenic spacer, partial sequence | Ellesmere Island | Strunecký *et al*., 2012b |  |
| JQ249765 | Uncultured cyanobacterium clone CBS1-61 16S ribosomal RNA gene, partial sequence | Ward Hunt Island | Lionard *et al*., 2012 |  |
| JQ249766 | Uncultured cyanobacterium clone CBS1-68 16S ribosomal RNA gene, partial sequence | Ward Hunt Island | Lionard *et al*., 2012 |  |
| JQ249774 | Uncultured cyanobacterium clone CBS31-D11 16S ribosomal RNA gene, partial sequence | Ward Hunt Island | Lionard *et al*., 2012 |  |
| JQ249779 | Uncultured cyanobacterium clone CBS31-F12 16S ribosomal RNA gene, partial sequence | Ward Hunt Island | Lionard *et al*., 2012 |  |
| JQ249786 | Uncultured cyanobacterium clone CBS32-D2 16S ribosomal RNA gene, partial sequence | Ward Hunt Island | Lionard *et al*., 2012 |  |
| JQ249787 | Uncultured cyanobacterium clone CBS32-D7 16S ribosomal RNA gene, partial sequence | Ward Hunt Island | Lionard *et al*., 2012 |  |
| JQ249793 | Uncultured cyanobacterium clone CBS32-F6 16S ribosomal RNA gene, partial sequence | Ward Hunt Island | Lionard *et al*., 2012 |  |
| JQ249794 | Uncultured cyanobacterium clone CBS32-G11 16S ribosomal RNA gene, partial sequence | Ward Hunt Island | Lionard *et al*., 2012 |  |
| JQ249802 | Uncultured cyanobacterium clone CBS2-08 16S ribosomal RNA gene, partial sequence | Ward Hunt Island | Lionard *et al*., 2012 |  |
| JQ249807 | Uncultured cyanobacterium clone CBS2-27 16S ribosomal RNA gene, partial sequence | Ward Hunt Island | Lionard *et al*., 2012 |  |
| JQ249821 | Uncultured cyanobacterium clone CBS2-66 16S ribosomal RNA gene, partial sequence | Ward Hunt Island | Lionard *et al*., 2012 |  |
| JQ310206 | Uncultured cyanobacterium clone D_ORG_D.5.6_C09-T7 16S ribosomal RNA gene, partial sequence | Baffin Island | Kleinteich *et al*., 2013 |  |
| JQ310245 | Uncultured cyanobacterium clone C_ORG_C.1.1_D05-T7 16S ribosomal RNA gene, partial sequence | Baffin Island | Kleinteich *et al*., 2013 |  |
| JQ310280 | Uncultured cyanobacterium clone A_ORG_A.4.2_C02-T7 16S ribosomal RNA gene, partial sequence | Baffin Island | Kleinteich *et al*., 2013 |  |

Supplementary Table 2: Northern high latitude and Arctic sequences

| Accession | Description | Location | Reference |  |
| --- | --- | --- | --- | --- |
| JQ769114 | Phormidium autumnale sv27 16S ribosomal RNA gene and 16S-23S ribosomal RNA intergenic spacer, partial sequence | Svalbard | Strunecký *et al*.,  2012b |  |
| JQ769116 | Phormidium autumnale sv12 16S ribosomal RNA gene and 16S-23S ribosomal RNA intergenic spacer, partial sequence | Svalbard | Strunecký *et al*., 2012b |  |
| JQ769118 | Phormidium autumnale sv22 16S ribosomal RNA gene and 16S-23S ribosomal RNA intergenic spacer, partial sequence | Svalbard | Strunecký *et al*., 2012b |  |
| JQ769119 | Phormidium autumnale sv26 16S ribosomal RNA gene and 16S-23S ribosomal RNA intergenic spacer, partial sequence | Svalbard | Strunecký *et al*., 2012b |  |
| JQ769120 | Phormidium autumnale svv4 16S ribosomal RNA gene and 16S-23S ribosomal RNA intergenic spacer, partial sequence | Svalbard | Strunecký *et al*., 2012b |  |
| JQ769121 | Phormidium autumnale sv09 16S ribosomal RNA gene and 16S-23S ribosomal RNA intergenic spacer, partial sequence | Svalbard | Strunecký *et al*., 2012b |  |
| JQ769126 | Phormidium autumnale s36 16S ribosomal RNA gene and 16S-23S ribosomal RNA intergenic spacer, partial sequence | Svalbard | Strunecký *et al*., 2012b |  |
| JQ769127 | Phormidium autumnale s33 16S ribosomal RNA gene and 16S-23S ribosomal RNA intergenic spacer, partial sequence | Svalbard | Strunecký *et al*., 2012b |  |
| JQ769128 | Phormidium autumnale svS1 16S ribosomal RNA gene and 16S-23S ribosomal RNA intergenic spacer, partial sequence | Svalbard | Strunecký *et al*., 2012b |  |
| JQ769130 | Phormidium autumnale sv30 16S ribosomal RNA gene and 16S-23S ribosomal RNA intergenic spacer, partial sequence | Svalbard | Strunecký *et al*., 2012b |  |
| JQ769133 | Phormidium autumnale sv25 16S ribosomal RNA gene and 16S-23S ribosomal RNA intergenic spacer, partial sequence | Svalbard | Strunecký *et al*., 2012b |  |
| JQ769134 | Phormidium autumnale svv11 16S ribosomal RNA gene and 16S-23S ribosomal RNA intergenic spacer, partial sequence | Svalbard | Strunecký *et al*., 2012b |  |
| JQ769138 | Phormidium autumnale sv11 16S ribosomal RNA gene and 16S-23S ribosomal RNA intergenic spacer, partial sequence | Svalbard | Strunecký *et al*., 2012b |  |
| JX887885 | Uncultured cyanobacterium clone Emix4.10 16S ribosomal RNA gene and 16S-23S ribosomal RNA intergenic spacer, partial sequence; and tRNA-Ile gene, complete sequence | Baffin Island | Kleinteich *et al*., 2013 |  |
| JX887886 | Uncultured cyanobacterium clone Emix1.11 16S ribosomal RNA gene and 16S-23S ribosomal RNA intergenic spacer, partial sequence; and tRNA-Ile gene, complete sequence | Baffin Island | Kleinteich *et al*., 2013 |  |
| JX887887 | Uncultured cyanobacterium clone A.4.11 16S ribosomal RNA gene and 16S-23S ribosomal RNA intergenic spacer, partial sequence; and tRNA-Ile gene, complete sequence | Baffin Island | Kleinteich *et al*., 2013 |  |
| JX887889 | Uncultured cyanobacterium clone Emix4.11 16S ribosomal RNA gene and 16S-23S ribosomal RNA intergenic spacer, partial sequence; and tRNA-Ile gene, complete sequence | Baffin Island | Kleinteich *et al*., 2013 |  |
| JX887890 | Uncultured cyanobacterium clone A10_3.8 16S ribosomal RNA gene and 16S-23S ribosomal RNA intergenic spacer, partial sequence; and tRNA-Ile gene, complete sequence | Baffin Island | Kleinteich *et al*., 2013 |  |
| JX887891 | Uncultured cyanobacterium clone A10_3.6 16S ribosomal RNA gene and 16S-23S ribosomal RNA intergenic spacer, partial sequence; and tRNA-Ile gene, complete sequence | Baffin Island | Kleinteich *et al*., 2013 |  |
| JX887892 | Uncultured cyanobacterium clone Emix3.12 16S ribosomal RNA gene and 16S-23S ribosomal RNA intergenic spacer, partial sequence; and tRNA-Ile gene, complete sequence | Baffin Island | Kleinteich *et al*., 2013 |  |
| JX887893 | Uncultured cyanobacterium clone E_Isolat_6 16S ribosomal RNA gene and 16S-23S ribosomal RNA intergenic spacer, partial sequence; and tRNA-Ile gene, complete sequence | Baffin Island | Kleinteich *et al*., 2013 |  |
| JX887894 | Uncultured cyanobacterium clone A10_3.4 16S ribosomal RNA gene and 16S-23S ribosomal RNA intergenic spacer, partial sequence; and tRNA-Ile gene, complete sequence | Baffin Island | Kleinteich *et al*., 2013 |  |
| JX887895 | Uncultured cyanobacterium clone Emix2.4 16S ribosomal RNA gene and 16S-23S ribosomal RNA intergenic spacer, partial sequence; and tRNA-Ile gene, complete sequence | Baffin Island | Kleinteich *et al*., 2013 |  |
| KF208379 | Pseudanabaena sp. lw0831 16S ribosomal RNA gene, partial sequence | Svalbard | Zhang, N/A |  |
| HM572425 | Uncultured bacterium clone Rock10 16S ribosomal RNA gene, partial sequence | Iceland | Cockell *et al*., 2011 |  |

Supplementary Table 3: Southern high latitude and Antarctic sequences

| Accession | Description | Location | Reference |
| --- | --- | --- | --- |
| AB098071 | Nostoc commune gene for 16S rRNA, partial sequence, strain:SO-42 | Showa Station | Arima *et al*., 2012 |
| AB519661 | Uncultured bacterium gene for 16S rRNA, partial sequence, clone: W51 | Langhovde | Fujii *et al*., 2010 |
| AB630384 | Uncultured bacterium gene for 16S ribosomal RNA, partial sequence, clone: MPB1-2 | Skarvsnes | Nakai *et al*., 2012 |
| AB630387 | Uncultured bacterium gene for 16S ribosomal RNA, partial sequence, clone: MPB1-5 | Skarvsnes | Nakai *et al*., 2012 |
| AB630388 | Uncultured bacterium gene for 16S ribosomal RNA, partial sequence, clone: MPB1-6 | Skarvsnes | Nakai *et al*., 2012 |
| AB630391 | Uncultured bacterium gene for 16S ribosomal RNA, partial sequence, clone: MPB1-9 | Skarvsnes | Nakai *et al*., 2012 |
| AB630681 | Uncultured bacterium gene for 16S ribosomal RNA, partial sequence, clone: MPB2-5 | Skarvsnes | Nakai *et al*., 2012 |
| AB630684 | Uncultured bacterium gene for 16S ribosomal RNA, partial sequence, clone: MPB2-8 | Skarvsnes | Nakai *et al*., 2012 |
| AB630686 | Uncultured bacterium gene for 16S ribosomal RNA, partial sequence, clone: MPB2-10 | Skarvsnes | Nakai *et al*., 2012 |
| AF076157 | Uncultured Antarctic bacterium LB3-1 16S ribosomal RNA gene, partial sequence | McMurdo Dry Valleys | Priscu *et al*., 1998 |
| AF076158 | Uncultured Antarctic bacterium LB3-76 16S ribosomal RNA gene, partial sequence | McMurdo Dry Valleys | Priscu *et al*., 1998 |
| AF076159 | Uncultured Antarctic bacterium LB3-53 16S ribosomal RNA gene, partial sequence | McMurdo Dry Valleys | Priscu *et al*., 1998 |
| AF076162 | Uncultured Antarctic bacterium LB3-80 16S ribosomal RNA gene, partial sequence | McMurdo Dry Valleys | Priscu *et al*., 1998 |
| AF076163 | Uncultured Antarctic bacterium LB3-47 16S ribosomal RNA gene, partial sequence | McMurdo Dry Valleys | Priscu *et al*., 1998 |
| AF076164 | Uncultured Antarctic bacterium LB3-75 16S ribosomal RNA gene, partial sequence | McMurdo Dry Valleys | Priscu *et al*., 1998 |
| AF098370 | Synechococcus-like str. ACE 16S ribosomal RNA gene, partial sequence | Vestfold Hills | Vincent *et al*., 2000 |
| AF098371 | Synechococcus-like str. PENDANT 16S ribosomal RNA gene, partial sequence | Vestfold Hills | Vincent *et al*., 2000 |
| AF098372 | Synechococcus-like str. ABRAXAS 16S ribosomal RNA gene, partial sequence | Vestfold Hills | Vincent *et al*., 2000 |
| AF170757 | LPP-group cyanobacterium QSSC5cya 16S ribosomal RNA gene, partial sequence | Vestfold Hills | Smith *et al*., 2000 |
| AF170758 | LPP-group cyanobacterium QSSC8cya 16S ribosomal RNA gene, partial sequence | Vestfold Hills | Smith *et al*., 2000 |
| AF170760 | Uncultured cyanobacterium QSSC5-B 16S ribosomal RNA gene, partial sequence | Vestfold Hills | Smith *et al*., 2000 |
| AF218373 | Microcoleus antarcticus UTCC 474 16S ribosomal RNA gene, partial sequence | McMurdo Ice Shelf | Casamatta *et al*., 2005 |
| AF218374 | Microcoleus glaciei UTCC 475 16S ribosomal RNA gene, partial sequence | McMurdo Ice Shelf | Casamatta *et al*., 2005 |
| AF218375 | Phormidium lumbricale UTCC 476 16S ribosomal RNA gene, partial sequence | McMurdo Ice Shelf | Casamatta *et al*., 2005 |
| AF263333 | Oscillatoria sp. Ant-G16 16S ribosomal RNA gene, partial sequence | Bratina Island | Nadeau *et al*., 2001 |
| AF263334 | Oscillatoria sp. Ant-G17 16S ribosomal RNA gene, partial sequence | Bratina Island | Nadeau *et al*., 2001 |
| AF263335 | Phormidium sp. Ant-Lunch 16S ribosomal RNA gene, partial sequence | Bratina Island | Nadeau *et al*., 2001 |
| AF263336 | Phormidium sp. Ant-Orange 16S ribosomal RNA gene, partial sequence | Bratina Island | Nadeau *et al*., 2001 |
| AF263337 | Oscillatoria sp. Ant-Salt 16S ribosomal RNA gene, partial sequence | Bratina Island | Nadeau *et al*., 2001 |
| AY151721 | Uncultured Antarctic cyanobacterium clone BGC-Fr032 16S ribosomal RNA gene, complete sequence | Lake Fryxell | Taton *et al*., 2003 |
| AY151722 | Uncultured Antarctic cyanobacterium clone BGC-Fr054 16S ribosomal RNA gene, complete sequence | Lake Fryxell | Taton *et al*., 2003 |
| AY151726 | Uncultured Antarctic cyanobacterium clone Fr048 16S ribosomal RNA gene, complete sequence | Lake Fryxell | Taton *et al*., 2003 |

Supplementary Table 3 (continued): Southern high latitude and Antarctic sequences

| Accession | Description | Location | Reference |
| --- | --- | --- | --- |
| AY151727 | Uncultured Antarctic cyanobacterium clone Fr094 16S ribosomal RNA gene, complete sequence | Lake Fryxell | Taton *et al*., 2003 |
| AY151730 | Uncultured Antarctic cyanobacterium clone Fr132 16S ribosomal RNA gene, complete sequence | Lake Fryxell | Taton *et al*., 2003 |
| AY151733 | Uncultured Antarctic cyanobacterium clone Fr297 16S ribosomal RNA gene, complete sequence | Lake Fryxell | Taton *et al*., 2003 |
| AY151734 | Uncultured Antarctic cyanobacterium clone Fr304 16S ribosomal RNA gene, complete sequence | Lake Fryxell | Taton *et al*., 2003 |
| AY250870 | Uncultured cyanobacterium clone FBP256 16S ribosomal RNA gene, partial sequence | McMurdo Dry Valleys | de la Torre, *et al*., 2003 |
| AY493572 | Leptolyngbya antarctica ANT.L67.1 16S ribosomal RNA gene, partial sequence | McMurdo Dry Valleys | Taton *et al*., 2006a |
| AY493578 | Phormidesmis priestleyi ANT.L52.4 16S ribosomal RNA gene, partial sequence | McMurdo Dry Valleys | Taton *et al*., 2006a |
| AY493579 | Phormidesmis priestleyi ANT.L52.6 16S ribosomal RNA gene, partial sequence | McMurdo Dry Valleys | Taton *et al*., 2006a |
| AY493580 | Phormidesmis priestleyi ANT.LG2.4 16S ribosomal RNA gene, partial sequence | McMurdo Dry Valleys | Taton *et al*., 2006a |
| AY493581 | Phormidesmis priestleyi ANT.L66.1 16S ribosomal RNA gene, partial sequence | McMurdo Dry Valleys | Taton *et al*., 2006a |
| AY493582 | Phormidesmis priestleyi ANT.L61.2 16S ribosomal RNA gene, partial sequence | McMurdo Dry Valleys | Taton *et al*., 2006a |
| AY493583 | Plectolyngbya hodgsonii ANT.LPR2.2 16S ribosomal RNA gene, partial sequence | McMurdo Dry Valleys | Taton *et al*., 2006a |
| AY493584 | Leptolyngbya sp. ANT.L52.1 16S ribosomal RNA gene, partial sequence | McMurdo Dry Valleys | Taton *et al*., 2006a |
| AY493585 | Phormidesmis priestleyi ANT.LPR2.6 16S ribosomal RNA gene, partial sequence | McMurdo Dry Valleys | Taton *et al*., 2006a |
| AY493586 | Phormidesmis priestleyi ANT.LACV5.1 16S ribosomal RNA gene, partial sequence | McMurdo Dry Valleys | Taton *et al*., 2006a |
| AY493588 | Leptolyngbya antarctica ANT.LAC.1 16S ribosomal RNA gene, partial sequence | McMurdo Dry Valleys | Taton *et al*., 2006a |
| AY493590 | Leptolyngbya antarctica ANT.BFI.1 16S ribosomal RNA gene, partial sequence | McMurdo Dry Valleys | Taton *et al*., 2006a |
| AY493591 | Nostoc sp. ANT.L34.1 16S ribosomal RNA gene, partial sequence | McMurdo Dry Valleys | Taton *et al*., 2006a |
| AY493592 | Nostoc sp. ANT.L61.1 16S ribosomal RNA gene, partial sequence | McMurdo Dry Valleys | Taton *et al*., 2006a |
| AY493593 | Nostoc sp. ANT.L52B.8 16S ribosomal RNA gene, partial sequence | McMurdo Dry Valleys | Taton *et al*., 2006a |
| AY493594 | Nostoc sp. ANT.L52B.1 16S ribosomal RNA gene, partial sequence | McMurdo Dry Valleys | Taton *et al*., 2006a |
| AY493595 | Nostoc sp. ANT.LG2.6 16S ribosomal RNA gene, partial sequence | McMurdo Dry Valleys | Taton *et al*., 2006a |
| AY493596 | Coleodesmium sp. ANT.L52B.5 16S ribosomal RNA gene, partial sequence | McMurdo Dry Valleys | Taton *et al*., 2006a |
| AY493597 | Calothrix sp. ANT.LPR2.4 16S ribosomal RNA gene, partial sequence | McMurdo Dry Valleys | Taton *et al*., 2006a |
| AY493598 | Phormidium murrayii ANT.LPE.2 16S ribosomal RNA gene, partial sequence | McMurdo Dry Valleys | Taton *et al*., 2006a |
| AY493599 | Chondrocystis sp. ANT.L59B.1 16S ribosomal RNA gene, partial sequence | McMurdo Dry Valleys | Taton *et al*., 2006a |
| AY493600 | Phormidium pseudopriestleyi ANT.LACV5.3 16S ribosomal RNA gene, partial sequence | McMurdo Dry Valleys | Taton *et al*., 2006a |
| AY493615 | Plectolyngbya hodgsonii ANT.LG2.1 16S ribosomal RNA gene, partial sequence | McMurdo Dry Valleys | Taton *et al*., 2006a |
| AY493627 | Phormidium murrayii ANT.LACV5.2 16S ribosomal RNA gene, partial sequence | McMurdo Dry Valleys | Taton *et al*., 2006a |
| AY541548 | Uncultured Antarctic cyanobacterium clone OraP15 16S ribosomal RNA gene, partial sequence | McMurdo ice Shelf | Jungblut *et al*., 2005 |
| AY541554 | Uncultured Antarctic cyanobacterium clone FreP31 16S ribosomal RNA gene, partial sequence | McMurdo Ice Shelf | Jungblut *et al*., 2005 |

Supplementary Table 3 (continued): Southern high latitude and Antarctic sequences

| \| Accession \| Description \| Location \| Reference \| \| --- \| --- \| --- \| --- \| \| AY541565 \| Uncultured Antarctic cyanobacterium clone FreP07 16S ribosomal RNA gene, partial sequence \| McMurdo Ice Shelf \| Jungblut *et al*., 2005 \| \| DQ015808 \| Uncultured bacterium clone ELB16-090 16S ribosomal RNA gene, complete sequence \| McMurdo Dry Valleys \| Glatz *et al*., 2006 \| \| DQ181668 \| Uncultured cyanobacterium clone A132 16S ribosomal RNA gene, complete sequence \| Vestfold Hills \| Taton *et al*., 2006b \| \| DQ181669 \| Uncultured cyanobacterium clone A180 16S ribosomal RNA gene, complete sequence \| Vestfold Hills \| Taton *et al*., 2006b \| \| DQ181670 \| Uncultured cyanobacterium clone A191 16S ribosomal RNA gene, complete sequence \| Vestfold Hills \| Taton *et al*., 2006b \| \| DQ181672 \| Uncultured cyanobacterium clone RD010 16S ribosomal RNA gene, complete sequence \| Larsemann Hills \| Taton *et al*., 2006b \| \| DQ181677 \| Uncultured cyanobacterium clone RD107 16S ribosomal RNA gene, complete sequence \| Larsemann Hills \| Taton *et al*., 2006b \| \| DQ181678 \| Uncultured cyanobacterium clone RJ010 16S ribosomal RNA gene, complete sequence \| Larsemann Hills \| Taton *et al*., 2006b \| \| DQ181680 \| Uncultured cyanobacterium clone RJ045 16S ribosomal RNA gene, complete sequence \| Larsemann Hills \| Taton *et al*., 2006b \| \| DQ181682 \| Uncultured cyanobacterium clone RJ094 16S ribosomal RNA gene, complete sequence \| Larsemann Hills \| Taton *et al*., 2006b \| \| DQ181683 \| Uncultured cyanobacterium clone RJ096 16S ribosomal RNA gene, complete sequence \| Larsemann Hills \| Taton *et al*., 2006b \| \| DQ181686 \| Uncultured cyanobacterium clone H-B02 16S ribosomal RNA gene, complete sequence \| Larsemann Hills \| Taton *et al*., 2006b \| \| DQ181687 \| Uncultured cyanobacterium clone H-B07 16S ribosomal RNA gene, complete sequence \| Larsemann Hills \| Taton *et al*., 2006b \| \| DQ181689 \| Uncultured cyanobacterium clone R8-B31 16S ribosomal RNA gene, complete sequence \| Raur Islands \| Taton *et al*., 2006b \| \| DQ181690 \| Uncultured cyanobacterium clone R8-R13 16S ribosomal RNA gene, complete sequence \| Raur Islands \| Taton *et al*., 2006b \| \| DQ181691 \| Uncultured cyanobacterium clone R8-R56 16S ribosomal RNA gene, complete sequence \| Raur Islands \| Taton *et al*., 2006b \| \| DQ181692 \| Uncultured cyanobacterium clone R8-R60 16S ribosomal RNA gene, complete sequence \| Raur Islands \| Taton *et al*., 2006b \| \| DQ181693 \| Uncultured cyanobacterium clone R8-R79 16S ribosomal RNA gene, complete sequence \| Raur Islands \| Taton *et al*., 2006b \| \| DQ181701 \| Uncultured cyanobacterium clone RD065 16S ribosomal RNA gene, partial sequence \| Larsemann Hills \| Taton *et al*., 2006b \| \| DQ181740 \| Uncultured cyanobacterium clone H-A07 16S ribosomal RNA gene, partial sequence \| Larsemann Hills \| Taton *et al*., 2006b \| \| DQ366018 \| Uncultured bacterium clone 20B9 16S ribosomal RNA gene, partial sequence \| McMurdo Dry Valleys \| Aislabie *et al*., 2006 \| \| DQ493872 \| Phormidium murrayi Ant-Ph58 16S ribosomal RNA gene, partial sequence \| Antarctic Peninsula \| Comte *et al*., 2007 \| \| DQ493874 \| Phormidium autumnale Ant-Ph68 16S ribosomal RNA gene, partial sequence \| South Orkney Islands \| Comte *et al*., 2007 \| \| DQ521473 \| Uncultured bacterium clone ANTLV1_B04 16S ribosomal RNA gene, partial sequence \| McMurdo Dry Valleys \| Mosier *et al*., 2007 \| \| DQ521499 \| Uncultured bacterium clone ANTLV1_H06 16S ribosomal RNA gene, partial sequence \| McMurdo Dry Valleys \| Mosier *et al*., 2007 \| \| DQ521500 \| Uncultured bacterium clone ANTLV1_H09 16S ribosomal RNA gene, partial sequence \| McMurdo Dry Valleys \| Mosier *et al*., 2007 \| \| DQ521503 \| Uncultured bacterium clone ANTLV2_A01 16S ribosomal RNA gene, partial sequence \| McMurdo Dry Valleys \| Mosier *et al*., 2007 \| \| DQ521509 \| Uncultured bacterium clone ANTLV2_C09 16S ribosomal RNA gene, partial sequence \| McMurdo Dry Valleys \| Mosier *et al*., 2007 \| \| EU852489 \| Uncultured Antarctic cyanobacterium clone TM2FOCH1 16S ribosomal RNA gene, partial sequence \| Transantarctic Mountains \| Fernández-Carazo *et al*., 2011 \| \| EU852490 \| Uncultured Antarctic cyanobacterium clone TM3FOCB5 16S ribosomal RNA gene, partial sequence \| Transantarctic Mountains \| Fernández-Carazo *et al*., 2011 \| |  |  |  |
| --- | --- | --- | --- | --- | --- | --- | --- | --- | --- | --- | --- | --- | --- | --- | --- | --- | --- | --- | --- | --- | --- | --- | --- | --- | --- | --- | --- | --- | --- | --- | --- | --- | --- | --- | --- | --- | --- | --- | --- | --- | --- | --- | --- | --- | --- | --- | --- | --- | --- | --- | --- | --- | --- | --- | --- | --- | --- | --- | --- | --- | --- | --- | --- | --- | --- | --- | --- | --- | --- | --- | --- | --- | --- | --- | --- | --- | --- | --- | --- | --- | --- | --- | --- | --- | --- | --- | --- | --- | --- | --- | --- | --- | --- | --- | --- | --- | --- | --- | --- | --- | --- | --- | --- | --- | --- | --- | --- | --- | --- | --- | --- | --- | --- | --- | --- | --- | --- | --- | --- | --- | --- | --- | --- | --- | --- | --- | --- |

Supplementary Table 3 (continued): Southern high latitude and Antarctic sequences

| Accession | Description | Location | Reference |
| --- | --- | --- | --- |
| EU852498 | Phormidium murrayi TM2ULC130 16S ribosomal RNA gene, partial sequence | Transantarctic Mountains | Fernández-Carazo *et al*., 2011 |
| FJ805913 | Uncultured Chroococcidiopsis sp. clone AN1_1 16S ribosomal RNA gene, partial sequence; 16S-23S ribosomal RNA intergenic spacer, complete sequence; and 23S ribosomal RNA gene, partial sequence | McMurdo Dry Valleys | Bahl *et al*., 2011 |
| FJ805914 | Uncultured Chroococcidiopsis sp. clone AN1_2 16S ribosomal RNA gene, partial sequence; 16S-23S ribosomal RNA intergenic spacer, complete sequence; and 23S ribosomal RNA gene, partial sequence | McMurdo Dry Valleys | Bahl *et al*., 2011 |
| FN811215 | Uncultured cyanobacterium partial 16S rRNA gene, clone UMAB-cl-31 | Alexander Island | Chong *et al*., 2012 |
| FN811217 | Uncultured cyanobacterium partial 16S rRNA gene, clone UMAB-cl-33 | Alexander Island | Chong *et al*., 2012 |
| FN811218 | Uncultured cyanobacterium partial 16S rRNA gene, clone UMAB-cl-34 | Alexander Island | Chong *et al*., 2012 |
| FN811219 | Uncultured cyanobacterium partial 16S rRNA gene, clone UMAB-cl-35 | Alexander Island | Chong *et al*., 2012 |
| FN811228 | Uncultured cyanobacterium partial 16S rRNA gene, clone UMAB-cl-44 | Alexander Island | Chong *et al*., 2012 |
| FN811229 | Uncultured cyanobacterium partial 16S rRNA gene, clone UMAB-cl-45 | Alexander Island | Chong *et al*., 2012 |
| FN811234 | Uncultured cyanobacterium partial 16S rRNA gene, clone UMAB-cl-50 | Alexander Island | Chong *et al*., 2012 |
| FN811236 | Uncultured cyanobacterium partial 16S rRNA gene, clone UMAB-cl-52 | Alexander Island | Chong *et al*., 2012 |
| FN811237 | Uncultured cyanobacterium partial 16S rRNA gene, clone UMAB-cl-53 | Alexander Island | Chong *et al*., 2012 |
| FN811239 | Uncultured cyanobacterium partial 16S rRNA gene, clone UMAB-cl-55 | Alexander Island | Chong *et al*., 2012 |
| FN811246 | Uncultured cyanobacterium partial 16S rRNA gene, clone UMAB-cl-62 | Alexander Island | Chong *et al*., 2012 |
| FR749806 | Uncultured cyanobacterium partial 16S rRNA gene, clone UMAB-cl-181 | Alexander Island | Chong *et al*., 2012 |
| FR822750 | Calothrix elsteri partial 16S rRNA gene, strain CCALA 953 | James Ross Island | Komárek *et al*., 2012b |
| HF678504 | Phormidium sp. CCAP 1462/11 partial 16S rRNA gene | South Orkney Islands | Day *et al*., N/A |
| HM101226 | Nostoc commune ULC146 16S ribosomal RNA gene, partial sequence | Sor Rondane | Fernández-Carazo *et al*., 2012 |
| JN230327 | Phormidium autumnale JR1 16S ribosomal RNA gene and 16S-23S ribosomal RNA intergenic spacer, partial sequence | James Ross Island | Strunecký *et al*., 2012a |
| JN230332 | Phormidium autumnale JR20 16S ribosomal RNA gene and 16S-23S ribosomal RNA intergenic spacer, partial sequence | James Ross Island | Strunecký *et al*., 2012a |
| JN230333 | Phormidium autumnale JR2 16S ribosomal RNA gene and 16S-23S ribosomal RNA intergenic spacer, partial sequence | James Ross Island | Strunecký *et al*., 2012a |
| JN230334 | Phormidium autumnale JR5 16S ribosomal RNA gene and 16S-23S ribosomal RNA intergenic spacer, partial sequence | James Ross Island | Strunecký *et al*., 2012a |
| JN230337 | Phormidium autumnale JR4 16S ribosomal RNA gene and 16S-23S ribosomal RNA intergenic spacer, partial sequence | James Ross Island | Strunecký *et al*., 2012a |
| JN230338 | Phormidium autumnale JR3 16S ribosomal RNA gene and 16S-23S ribosomal RNA intergenic spacer, partial sequence | James Ross Island | Strunecký *et al*., 2012a |
| JN230339 | Phormidium autumnale JR6 16S ribosomal RNA gene and 16S-23S ribosomal RNA intergenic spacer, partial sequence | James Ross Island | Strunecký *et al*., 2012a |
| JN230346 | Phormidium autumnale KG29 16S ribosomal RNA gene and 16S-23S ribosomal RNA intergenic spacer, partial sequence | South Orkney Islands | Strunecký *et al*., 2012a |
| JN230347 | Phormidium autumnale KI19 16S ribosomal RNA gene and 16S-23S ribosomal RNA intergenic spacer, partial sequence | Killingbeck Island | Strunecký *et al*., 2012a |
| JN857999 | Uncultured cyanobacterium clone IBIS549 16S ribosomal RNA gene, partial sequence | Pyramid Trough | Jungblut *et al*., 2012 |
| JN858005 | Uncultured cyanobacterium clone TUT465 16S ribosomal RNA gene, partial sequence | Pyramid Trough | Jungblut *et al*., 2012 |
| JQ310427 | Cyanobacterium enrichment culture clone 3_4_3.6.5_C07-T7 16S ribosomal RNA gene, partial sequence | Byers Peninsula | Kleinteich *et al*., 2013 |

Supplementary Table 3 (continued): Southern high latitude and Antarctic sequences

| Accession | Description | Location | Reference |
| --- | --- | --- | --- |
| JQ310430 | Cyanobacterium enrichment culture clone 3_4_3.1.6_D05-T7 16S ribosomal RNA gene, partial sequence | Byers Peninsula | Kleinteich *et al*., 2013 |
| JQ687330 | Phormidium sp. CYN64 16S ribosomal RNA gene, partial sequence | Pyramid Trough | Martineau *et al*., 2013 |
| JQ687331 | Leptolyngbya antarctica CYN65 16S ribosomal RNA gene, partial sequence | Pyramid Trough | Martineau *et al*., 2013 |
| JQ687334 | Leptolyngbya sp. CYN68 16S ribosomal RNA gene, partial sequence | Pyramid Trough | Martineau *et al*., 2013 |
| JQ687335 | Phormidesmis priestleyi CYN71 16S ribosomal RNA gene, partial sequence | McMurdo Ice Shelf | Martineau *et al*., 2013 |
| JX559238 | Uncultured bacterium clone XXM_1_100 16S ribosomal RNA gene, partial sequence | South Orkney Islands | Xia *et al*., N/A |
| KC346265 | Hydrocoryne sp. CENA393 16S ribosomal RNA gene, partial sequence; 16S-23S ribosomal RNA intergenic spacer, tRNA-Ile and tRNA-Ala genes, complete sequence; and 23S ribosomal RNA gene, partial sequence | South Orkney Islands | Genuario *et al*., 2013 |
| KC346266 | Hydrocoryne sp. CENA398 16S ribosomal RNA gene, partial sequence; 16S-23S ribosomal RNA intergenic spacer, tRNA-Ile and tRNA-Ala genes, complete sequence; and 23S ribosomal RNA gene, partial sequence | South Orkney Islands | Genuario *et al*., 2013 |
| KC346267 | Hydrocoryne sp. UFV-ANT31 16S ribosomal RNA gene, partial sequence | South Orkney Islands | Genuario *et al*., 2013 |
| KC346268 | Hydrocoryne sp. UFV-ANT32 16S ribosomal RNA gene, partial sequence | South Orkney Islands | Genuario *et al*., 2013 |
| KF294471 | Uncultured cyanobacterium clone MVB103 16S ribosomal RNA gene, partial sequence | McMurdo Dry Valleys | Yung & Kong, 2012 |
| KF294472 | Uncultured cyanobacterium clone MVB77 16S ribosomal RNA gene, partial sequence | McMurdo Dry Valleys | Yung & Kong, 2012 |
| KF294483 | Uncultured cyanobacterium clone MVB86 16S ribosomal RNA gene, partial sequence | McMurdo Dry Valleys | Yung & Kong, 2012 |
| KF294485 | Uncultured cyanobacterium clone MVB1 16S ribosomal RNA gene, partial sequence | McMurdo Dry Valleys | Yung & Kong, 2012 |
| KF294486 | Uncultured cyanobacterium clone MVB287 16S ribosomal RNA gene, partial sequence | McMurdo Dry Valleys | Yung & Kong, 2012 |
| KF294487 | Uncultured cyanobacterium clone MVB234 16S ribosomal RNA gene, partial sequence | McMurdo Dry Valleys | Yung & Kong, 2012 |
| KF294488 | Uncultured cyanobacterium clone MVB59 16S ribosomal RNA gene, partial sequence | McMurdo Dry Valleys | Yung & Kong, 2012 |
| KF294489 | Uncultured cyanobacterium clone MVB52 16S ribosomal RNA gene, partial sequence | McMurdo Dry Valleys | Yung & Kong, 2012 |
| KF294490 | Uncultured cyanobacterium clone MVB9 16S ribosomal RNA gene, partial sequence | McMurdo Dry Valleys | Yung & Kong, 2012 |
| KF294491 | Uncultured cyanobacterium clone MVB107 16S ribosomal RNA gene, partial sequence | McMurdo Dry Valleys | Yung & Kong, 2012 |
| KF294492 | Uncultured cyanobacterium clone MVB185 16S ribosomal RNA gene, partial sequence | McMurdo Dry Valleys | Yung & Kong, 2012 |

**2. Supplementary Figures**

**Supplementary Figure 1:** Unconstrained phylogeny of cyanobacteria. Topology differs considerably when using 16SrRNA genes alone compared to that recovered using 16SrRNA genes with a genome constraint, particularly regarding the placement of *Pseudanabaena*, *Aphanocapsa* and *Chroococcidiopsis*.
