# Supplementary figures and images for: Multiple adaptations to polar and alpine environments within cyanobacteria: a phylogenomic and Bayesian approach
Source: Front Microbiol. 2015 Oct 13;6:1070. doi: 10.3389/fmicb.2015.01070 (PMC4602134; doi:10.3389/fmicb.2015.01070)

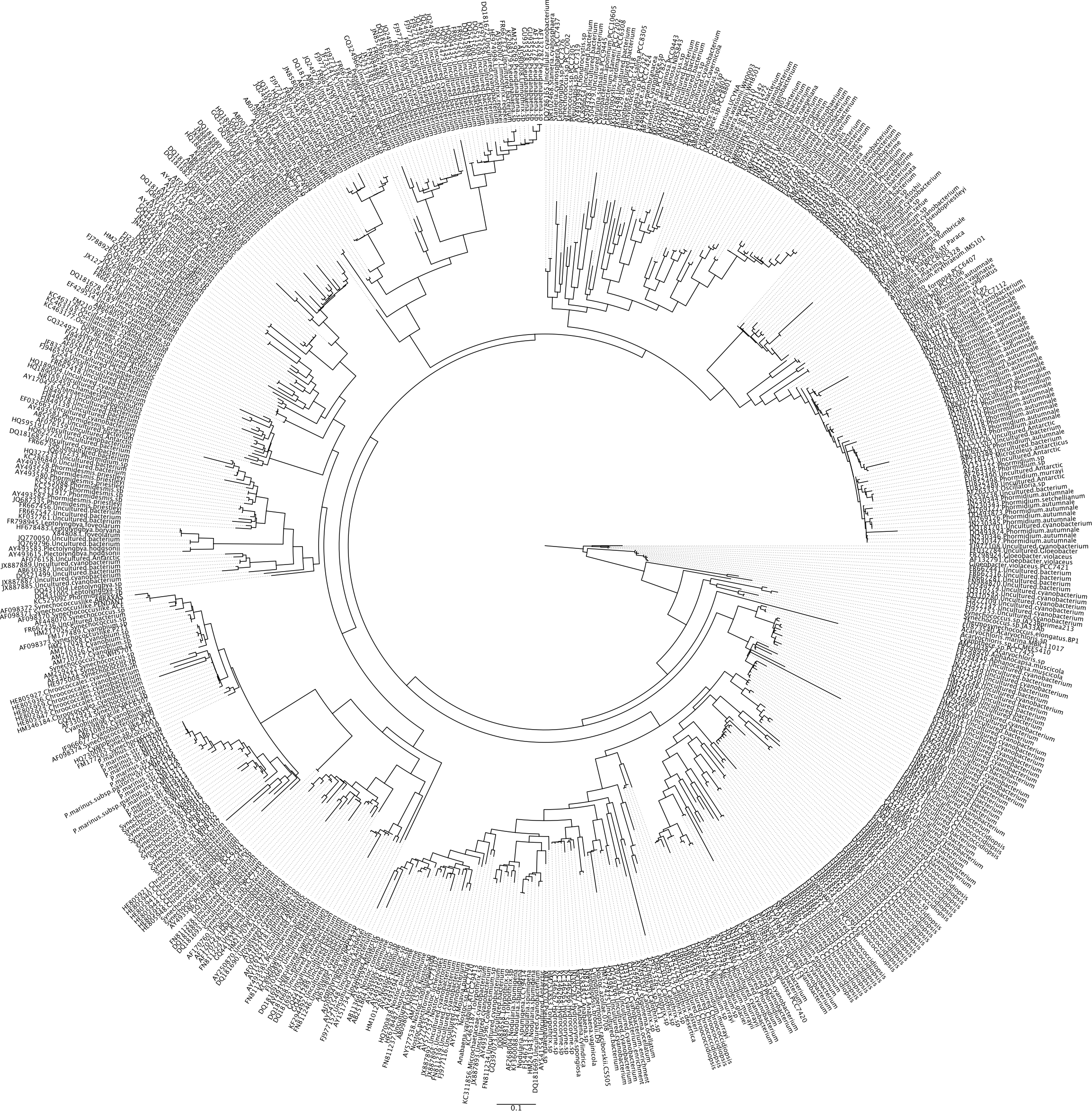

Supplement: Supplementary file 2 [file Image_1.TIFF]
